# Reciprocal Associations Between Parental Anxiety/Depression and Emotional/Behavioral Difficulties in Autistic Children Following Their Diagnosis

**DOI:** 10.1002/aur.70220

**Published:** 2026-03-19

**Authors:** Maëva Monnier, Hugo Peyre, Marianne Peries, Valentin Simoncic, Guillaume Nicolet, Cécile Michelon, Yashvin Seetahul, Amaria Baghdadli, the Elena Study Group

**Affiliations:** ^1^ Autism Resources Centre of Languedoc‐Roussillon & Centre of Excellence for Autism and Neurodevelopmental Disorders (CeAND) Montpellier University Hospital Montpellier France; ^2^ Developmental Psychiatry and Trajectories Team Centre for Research in Epidemiology and Population Health (CESP) Villejuif France; ^3^ Doctoral School of Public Health (EDSP) University of Paris‐Saclay Kremlin‐Bicêtre France; ^4^ University of Versailles‐Saint‐Quentin in Yvelines Versailles France; ^5^ Psychology Department University of Innsbruck Innsbruck Austria; ^6^ Faculty of Medicine University of Montpellier Montpellier France

**Keywords:** anxiety, autism, behavioral difficulties, children, depression, parents

## Abstract

Emotional and behavioral difficulties (EBD) are common in autistic children, while anxiety and depressive symptoms (ADS) are prevalent in their parents. However, the bidirectional relationship between the parents' and children's symptoms remains unclear, especially in the years following the child's autism diagnosis. Addressing this gap, our study investigates the bidirectional association between parental ADS and two subdomains of EBD (internalizing and externalizing difficulties) in autistic children from diagnosis (T0) to 3 years later (T1). Data from the French ELENA cohort were analyzed using two‐wave cross‐lagged panel models (CLPM). At the time of diagnosis, 55.2% of mothers and 42.7% of fathers among 315 parents exhibited clinically significant ADS, while 61.3% of children experienced clinical EBD. The CLPMs did not reveal any directional association between parental ADS and children's EBD. However, we observed significant autoregressive effects for parental ADS and children's EBD, with a moderate positive correlation between the two at the time of diagnosis. Our findings highlight significant psychological distress in both parents and children at the time of diagnosis and therefore recommend suitable interventions for families requiring social, financial, or psychological support. Future longitudinal studies with greater representation of girls, using continuous‐time models could clarify whether parent–child associations are time‐lag dependent (including identifying potential peak lags) and whether they differ across parent–child sex dyads.

## Lay Summary

Parents of autistic children often experience significant anxiety and depressive symptoms, while these children frequently face emotional and behavioral difficulties (EBD). Our French study shows that parental distress and children's EBD are moderately correlated at diagnosis, but these factors do not appear to influence each other 3 years later. These findings underscore the need for early support during the diagnostic period to address the psychological well‐being of both parents and children. However, because we did not find evidence that these difficulties influence one another 3 years after diagnosis, interventions should prioritize immediate coping strategies and adaptive resources rather than targeting long‐term reciprocal effects.

## 1 Introduction

Anxiety and depressive symptoms (ADS) are more prevalent and severe in parents of autistic children than in parents of neurotypical children (Almansour et al. 2013; Ansari et al. 2021). A meta‐analysis of 31 articles, including data from 9208 parents of autistic children, reveals that 31% (95% CI [0.24, 0.38]) of parents experience depressive disorders and 33% (95% CI [0.20, 0.48]) suffer from anxiety disorders (Schnabel et al. 2020). Prior research indicates that parental psychological distress appears to be more often correlated with the intensity of emotional and behavioral difficulties (EBD) in autistic children than other clinical characteristics such as the severity of autistic symptoms or adaptive skills (Davis and Carter 2008; Miniarikova et al. 2022; Zhou et al. 2019). Several cross‐sectional studies highlight this association in the context of autism (Falk et al. 2014; Khusaifan and El Keshky 2022; Lin et al. 2023, 2021; McRae et al. 2018; Nahar et al. 2022; Su et al. 2018; Yorke et al. 2018; Zhou et al. 2019). The child's EBD can be divided into two subdimensions, which are not‐mutually exclusive, called internalizing and externalizing difficulties (Achenbach 2011; Achenbach and Rescorla 2001). While internalizing difficulties are inner‐directed emotional and behavioral difficulties (e.g., anxiety, depression, social withdrawal, or somatic complaints), externalizing difficulties refer to difficulties directed outwards and can be perceived by others (e.g., impulsivity, hyperactivity, non‐compliance, or aggressiveness). Investigating the dynamics of the relationship between parental ADS and EBD in autistic children is of particular importance, given the higher frequency and severity observed in autistic children versus neurotypical children (Ding et al. 2021; May and Williams 2024; Totsika et al. 2013).

Several studies conducted in general population samples highlight a bidirectional relationship between parental ADS and children's EBD (Bagner et al. 2013; Hentges et al. 2021; May and Williams 2024; Thompson and Henrich 2022). These studies draw on the transactional model of development, suggesting that a child's development results from continuous and bidirectional interactions between the child and their environment, notably their family. However, due to the relatively low prevalence of autism, only four studies worldwide have been conducted within this context, resulting in limited sample sizes and contradictory results (May and Williams 2024; Piro‐Gambetti et al. 2023; Totsika et al. 2013), and the findings from these four studies are mixed. For example, a study of 132 autistic children in the United Kingdom did not demonstrate a bidirectional relationship between children's EBD and mothers' ADS (Totsika et al. 2013). Instead, they found that mothers' psychological distress is a risk factor for subsequent EBD. Using the same mental health measurement scales, an Australian study involving 409 children—including 164 with attention‐deficit/hyperactivity disorder, 159 autistic children, and 86 autistic children with co‐occurring ADHD—demonstrated a bidirectional association between mothers' psychological distress and children's EBD (May and Williams 2024). This bidirectional link was highlighted in children between the ages of 6 and 10, which corresponds to the average age of autism (6 years) and ADHD (9.1 years) diagnoses in this study. The authors suggested that the bidirectional association may be particularly evident around the time of the child's diagnosis since obtaining a diagnosis could potentially lead to significant stress for families (Bonis 2016; May and Williams 2024; Vernhet et al. 2019). Another study in the United States which included 188 autistic children examined the relationships between parental depressive symptoms and internalizing difficulties in autistic children over four annual measurements (Piro‐Gambetti et al. 2023) and found a bidirectional association between internalizing difficulties in autistic children and mothers' depression. To our knowledge, this is the only longitudinal study that has investigated the bidirectional relationship between fathers' depressive symptoms and internalizing difficulties in autistic children (Piro‐Gambetti et al. 2023). Their findings indicated a unidirectional association in which fathers' depression predicts children's subsequent internalizing difficulties. The authors suggested that mothers' depression may be more influential and exert a stronger impact on the children's internalizing difficulties than fathers' depression. There is a notable lack of research on fathers, partly attributable to their relatively low participation rates in studies, which leaves areas in this field that require deeper investigation.

Moreover, while the association between mother's ADS and externalizing difficulties in children has been researched in the general population (Song et al. 2022), this has not been studied specifically within the autism context. Hence, this study aims to explore the bidirectional association between parental ADS and EBD in autistic children from diagnosis to 3 years later, a period often marked by increased family distress (Lerthattasilp et al. 2015; Miniarikova et al. 2022). We hypothesized that: (i) mothers' ADS and children's EBD would be correlated at diagnosis (T0), (ii) mothers' ADS at T0 would predict children's EBD 3 years after (T1), (iii) children's EBD at T0 would predict mothers' ADS at T1. We investigated bidirectional relationships between mothers' ADS and two subdimensions of the children's EBD: internalizing and externalizing difficulties. Due to the limited data available on fathers (except Piro‐Gambetti et al. 2023), we looked into the relationship between fathers' ADS and children's EBD in an exploratory approach. Our study, including exploratory and sensitivity analyses, informs targeted recommendations for healthcare professionals and future research. The hypotheses and the analysis plan were preregistered on OSF (*osf.io/8de75*). Consistent with the autism language guide (Autism Alliance of Canada 2025), this paper employs identity‐first language (e.g., autistic children) to ensure terminology that is respectful, and devoid of ableist or stigmatizing connotations.

## 2 Methods

### 2.1 Participants

**FIGURE 1:**
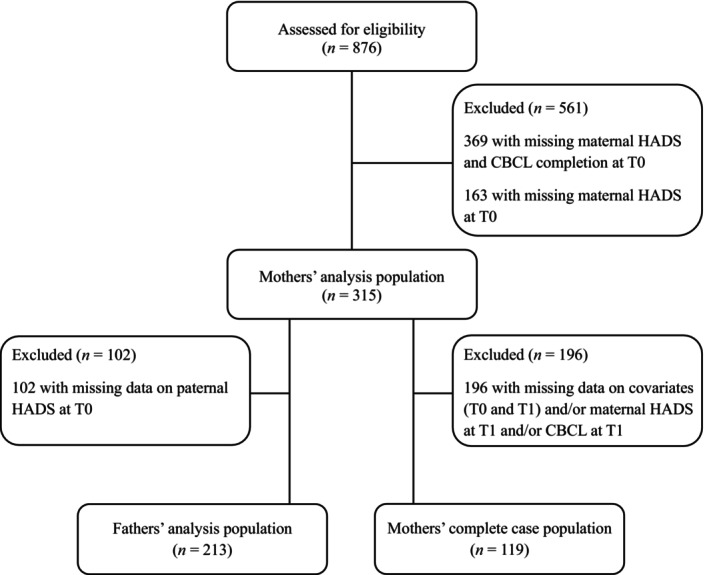
Flowchart illustrating participant selection for the study populations. CBCL, child behavior checklist; HADS, hospital anxiety and depression scale; T0, time of the child's autism diagnosis; T1, 3 years after T0.

The data were drawn from the ELENA cohort, a French prospective, longitudinal, and multiregional observational study involving 876 autistic children diagnosed aged 2–16 years. Children were included at the time of their first autism diagnosis, confirmed through a multidisciplinary assessment. The assessment was based on DSM‐5 (Diagnostic and Statistical Manual of Mental Disorders, Fifth Edition) criteria, using the ADOS‐2 (Autism Diagnostic Observation Schedule, Second Edition), the ADI‐R (Autism Diagnostic Interview‐Revised), the VABS‐II (Vineland Adaptive Behavior Scales, Second Edition), and psychological examinations measuring Intellectual Quotient (IQ, Baghdadli et al. 2019, 2020). Written parental consent and French language proficiency were also required. Data were collected at the time of the child's autism diagnosis (T0) and 3 years later (T1). Parental and child mental health, clinical and demographic data were collected. Inclusion criteria for the main analyses required completion of the hospital anxiety‐depression scale (HADS) by the mother and the child behavior checklist (CBCL) for the children at T0. The selection of study populations is described in the flowchart (see Figure 1).

### 2.2 Measures

#### 2.2.1 Parental Anxiety and/or Depressive Symptoms

Parental ADS were evaluated using the hospital anxiety and depression scale (HADS) at both T0 and T1 (Zigmond and Snaith 2013). The HADS is a reliable and valid tool for assessing depression and anxiety in both the general population and parents of autistic children (Almansour et al. 2013). It comprises 14 items, with seven addressing depression and seven relating to anxiety, each scored from 0 to 3. Total and subscale scores were computed, with higher scores indicating greater psychological distress. Cutoff points were set at 0–7 for no anxiety or depression, 8–10 for suspected symptoms, and 11–21 for significant symptoms of anxiety or depression. Total scores of 0–14 indicated absence of anxiety and depression while 15–42 indicated significant symptoms. The HADS demonstrates good internal consistency and construct validity (Bocéréan and Dupret 2014).

#### 2.2.2 Emotional and Behavioral Difficulties in Autistic Children

EBD were assessed using the child behavior checklist (CBCL) at both time points (Achenbach 2011; Achenbach and Rescorla 2017, 2001). Parents completed either the preschool version (CBCL/1.5–5, for ages 1.5–5) comprising 99 items or the school‐age version (CBCL/6–18, for ages 6–18) containing 112 items, based on their child's age. Responses ranged from (0) *not true* to (2) *very or often true*. Scores were calculated for EBD, internalizing difficulties (including anxiety/depression, withdrawal/depression, and somatic complaints), and externalizing difficulties (including rule‐breaking behaviors and aggressive behaviors). The EBD total score corresponds to the sum of internalizing and externalizing difficulties, and a third domain covering other difficulties (e.g., social, thought and attention). Normalized scores (*T*‐scores) were interpreted as follows: < 60 as normal, 60–64 as borderline, and ≥ 64 as clinical. Both versions of the CBCL showed strong reliability in the autism context (Pandolfi et al. 2009, 2012, 2014). Although *T*‐scores place the two CBCL versions on a common mathematical metric, they do not, rule out heterotypic continuity (Petersen et al. 2020). Accordingly, we assessed construct validity invariance for internalizing, externalizing, and total difficulties using Petersen's six criterion framework (Petersen et al. 2020), following prior application in autistic cohorts (Richard et al. 2025). Our analyses confirmed the construct validity and measurement invariance of the internalizing, externalizing, and total difficulties domains across CBCL versions (1½–5 and 6–18), supporting their comparability over time and robustness for in CLPMs (see Supporting Information: Methods S1).

### 2.3 Covariates

Each covariate was selected based on the findings of the meta‐analysis conducted by Yorke et al. (2018) on factors linked to family mental health within the autism context, and we also considered the available data in the ELENA cohort. Several child‐level variables at diagnosis were collected, including autism symptoms severity assessed using the calibrated severity score (CSS) of the Autism Diagnostic Observation Schedule, Second Edition (ADOS‐2; Gotham et al. 2009; Hus et al. 2014; Hus and Lord 2014; Shumway et al. 2012), child's sex, age, and IQ. The child's IQ score was estimated based on the age‐appropriate psychometric scale using an algorithm provided in the Supporting Information: IQ Score Algorithm. Additionally, parental level of education (never or primary or secondary vs. post‐secondary) and age were considered.

### 2.4 Statistical Analysis

All continuous variables were *z*‐standardized to harmonize scale, correct for large differences in variance, and improve numerical stability prior to all analyses. The primary outcome was analyzed with cross‐lagged panel models (CLPM) using the *Lavaan* package (Rosseel 2012) and the *sem()* function to evaluate bidirectional association between mothers' ADS and EBD in autistic children. We also fit specific CLPMs between mothers' ADS and internalizing/externalizing difficulties. Preregistered CLPMs were adjusted for a literature‐based set of T0 covariates entered as simultaneous predictors of each outcome at T0 and T1: child's sex, age, IQ, autism symptom severity; mother's and father's age and educational level.

A correlation matrix summarized associations between maternal and paternal ADS and the child's EBD at T0 and T1. Univariate analyses between these covariates and the CLPM outcomes at T0 are reported in Table S1; details on statistical tests are provided in Supporting Information: Methods S2. To assess the adequacy of our preregistered models and detect potential overfitting, we estimated several simplified models (see Tables S5–S16): (1) a parsimonious covariate‐adjusted model (see Supporting Information: Methods S2 and Table S2); (2) an unadjusted base model; and models fixing to zero the residual covariance between the focal variables at (3) T1, (4) T0, or (5) both T0 and T1, plus (6) a sensitivity model adjusted only for CBCL version (T0/T1) and age at diagnosis. For model (1), parsimonious empirical CLPMs were estimated by retaining only covariates that prospectively predicted the T1 outcome while controlling for the corresponding T0 score (see Table S1). Model (6) was included to mitigate potential bias arising from using different CBCL versions over time (see Tables S11–S16). Additional information on the CLPM, the estimators used, model comparison and model adequacy assessment are available in Supporting Information: Methods S2.

A detailed comparison of family characteristics between children included versus excluded from the study populations is also provided in Tables S3 and S4. Sensitivity analyses for the primary and specific outcomes were also conducted on the mothers' complete case population. The mothers' complete case corresponds to children and mothers for whom CBCL, HADS and all covariates were completed at T0 and T1 (*n* = 119, see Figure 1). To formulate hypotheses for future research on fathers' data, exploratory analysis was employed to investigate the bidirectional relationship between fathers' ADS and EBD in autistic children using CLPM. Several other exploratory analyses were conducted as well. We examined the bidirectional association between EBD in autistic children and mothers' symptoms of anxiety and depression separately. To investigate changes in anxiety and depressive symptoms in parents as well as in children's EBD between the time of diagnosis and 3 years later, exploratory analyses were performed using the paired samples Wilcoxon test for quantitative variables and McNemar tests or McNemar‐Bowker tests for qualitative variables. These analyses were carried out on the mothers' analysis population with paired measurements at the two time points.

All analyses were executed using SAS version 9.4 (SAS Institute Inc. 2023), R version 4.3.2 (R Core Team 2023), and R Studio (Posit Team 2024). Treatment of missing data in the CLPM is detailed in Supporting Information: Handling of missing data.

## 3 Results

### 3.1 Description of Children's and Parental Characteristics at Time of the Child's Diagnosis

The characteristics of the children and parents at time of the child's autism diagnosis (T0) are shown in Tables 1 and 2 (the distribution of these variables can be found in Figure S1). The mothers' analysis population is based on 315 included children with a mean age of 6.2 years (*±*3.5) and 80.0% boys (*n* = 252, see Table 1). Overall, their mean ADOS‐2 CSS score was 7.2 (±2.0) and mean IQ was 76.7 (±27.4) with 39.1% children having an IQ below 70. In this study, 61.3% (*n* = 193) of children had EBD in the clinical range at T0, and 12.1% (*n* = 38) were in the borderline range. Specifically, at T0, 59.7% of children were in the clinical range for internalizing difficulties, and 37.8% were in the clinical range for externalizing difficulties. Furthermore, among children in the clinical range for externalizing difficulties at T0, 84% (*n* = 100) were also in the clinical range for internalizing difficulties.

**TABLE 1:** Children's characteristics according to study populations at the time of the child's autism diagnosis.

	Mothers' analysis population (*n* = 315)				Mothers' complete case population (*n* = 119)			
	*N* (%)	*M* (SD)	Min–Max	NNa (%)^a^	*N* (%)	*M* (SD)	Min–Max	NNa (%)^a^
Sex				—				—
Boy	252 (80.00)				91 (76.47)			
Girl	63 (20.00)				28 (23.53)			
Age		6.16 (3.50)	2–16.59	—		5.92 (3.32)	2–15.99	—
IQ		76.69 (27.35)	20–138	—		77.68 (29.58)	20–138	—
IQ range				—				—
Profound disability	36 (11.43)				18 (15.13)			
Moderate disability	40 (12.70)				12 (10.08)			
Mild disability	47 (14.92)				14 (11.76)			
No disability	192 (60.95)				75 (63.03)			
ADOS‐CSS		7.18 (2.02)	1–10	10 (3.17)		7.03 (2.08)	1–10	—
SA‐CSS		7.16 (2.01)	2–10	8 (2.54)		6.83 (2.09)	2–10	—
RRB‐CSS		6.93 (2.27)	1–10	10 (3.17)		7.27 (2.11)	1–10	—
EBD total *T*‐score		64.92 (10.04)	30–87	—		65.12 (10.63)	30–86	—
EBD range				—				—
Clinical	193 (61.27)				78 (65.55)			
Borderline	38 (12.06)				11 (9.24)			
Normal	84 (26.67)				30 (25.21)			
Internalizing *T*‐score		64.44 (10.02)	29–88	—		64.36 (10.89)	29–85	—
Internalizing difficulties range				—				—
Clinical	188 (59.68)				77 (64.71)			
Borderline	43 (13.65)				11 (9.24)			
Normal	84 (26.67)				31 (26.05)			
Externalizing *T*‐score		60.12 (9.72)	28–85	—		60.56 (10.33)	28–85	—
Externalizing difficulties range				—				—
Clinical	119 (37.78)				47 (39.50)			
Borderline	46 (14.60)				20 (16.81)			
Normal	150 (47.62)				52 (43.70)			

Abbreviations: —, absence of missing data; ADOS, autism diagnostic observation schedule; CSS, calibrated severity score; EBD, emotional and behavioral difficulties; IQ, intellectual quotient; RRB, restricted and repetitive behaviors; SA, social affect.

^a^ Number of missing data per variable and associated percentage.

**TABLE 2:** Parental characteristics according to study populations at the time of the child's autism diagnosis.

	Mothers' analysis population (*n* = 315)				Mothers' complete case population (*n* = 119)			
	*N* (%)	*M* (SD)	Min–Max	NNa (%)^a^	*N* (%)	*M* (SD)	Min–Max	NNa (%)^a^
Mother's age		37.34 (6.34)	21–56	6 (1.90)		37.51 (5.84)	27–52	—
Mother's education level				17 (5.40)				—
Never or primary or secondary	107 (35.91)				38 (31.93)			
Post‐secondary	191 (64.09)				81 (68.07)			
Mother's total anxiety‐depression score		16.27 (6.78)	2–36	—		16.25 (6.47)	3–36	—
Mother's ADS				—				—
Significant ADS	174 (55.24)				67 (56.30)			
Absence of anxiety and depression	141 (44.76)				52 (43.70)			
Mother's anxiety level				—				—
Significant level of anxiety	126 (40.00)				47 (39.50)			
Suspected anxiety	93 (29.52)				34 (28.57)			
Absence of anxiety	96 (30.48)				38 (31.93)			
Mother's depression level				—				—
Significant level of depression	52 (16.51)				17 (14.29)			
Suspected depression	66 (20.95)				27 (22.69)			
Absence of depression	197 (62.54)				75 (63.03)			
Father's age		40.39 (7.57)	24–78	8 (2.54)		40.96 (7.49)	24–78	—
Father's education level				20 (6.35)				—
Never or primary or secondary	133 (45.08)				56 (47.06)			
Post‐secondary	162 (54.92)				63 (52.94)			
Father's total anxiety‐depression score		13.58 (6.60)	1–33	102 (32.38)		13.59 (6.26)	3–33	40 (33.61)
Father's ADS				102 (32.38)				40 (33.61)
Significant ADS	91 (42.72)				32 (40.51)			
Absence of anxiety and depression	122 (57.28)				47 (59.49)			
Father's anxiety level				99 (31.43)				38 (31.93)
Significant level of anxiety	51 (23.61)				19 (23.46)			
Suspected anxiety	58 (26.85)				24 (29.63)			
Absence of anxiety	107 (49.54)				38 (46.91)			
Father's depression level				97 (30.79)				38 (31.93)
Significant level of depression	24 (11.01)				9 (11.11)			
Suspected depression	40 (18.35)				18 (22.22)			
Absence of depression	154 (70.64)				54 (66.67)			

Abbreviations: —, absence of missing data; ADS, anxiety and/or depressive symptoms.

^a^ Number of missing data per variable and associated percentage.

In terms of parents, the average age for mothers at T0 was 37.3 (±6.3) and 40.4 for fathers (±7.6, see Table 2). Among mothers, 64.1% had an education level higher than secondary school, while the percentage for fathers was 54.9%. Concerning the mental health of parents, 55.2% of mothers exhibited significant ADS at time of the child's autism diagnosis, while fathers accounted for 42.7%. More specifically, 69.5% and 37.5% of mothers displayed suspected or significant symptoms of anxiety and depression, respectively. In contrast, 50.5% of fathers demonstrated suspected or significant symptoms of anxiety, while 29.4% experienced suspected or significant symptoms of depression.

The mothers' analysis population (*n* = 315) and the excluded population (*n* = 561) differed only in terms of the child's IQ, with excluded children showing a higher prevalence of disability (*p* = 0.002; see Table S3). This would suggest that children with lower IQs may require additional support, impacting parental availability and posing challenges for longitudinal follow‐up. Additionally, the mothers' complete case population (*n* = 119) and those excluded from the mothers' analysis population (*n* = 196) differed only in the severity of the child's social affect and restrictive repetitive behaviors (see Table S4).

### 3.2 Associations Between Covariates and Outcomes at T0 and T1

**FIGURE 2:**
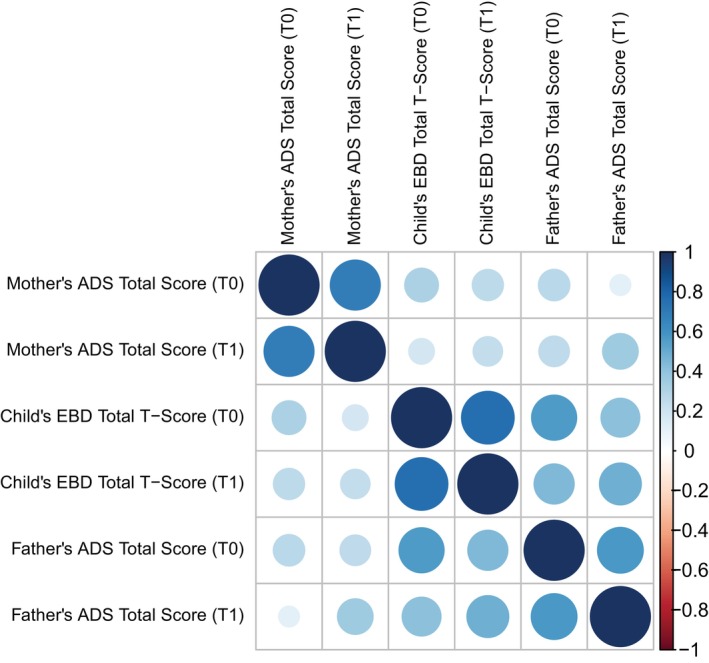
Correlations between child's emotional/behavioral difficulties (EBD) and mothers'/fathers' anxiety/depressive symptoms (ADS) at T0 and T1. T0, time of the child's autism diagnosis; T1, 3 years after T0.

Descriptive correlations among key variables are shown in Figure 2. Child's EBD total *T*‐score correlated with both mother's and father's ADS total scores at baseline (T0) and at the 3‐year follow‐up (T1). Concerning associations between covariates and outcomes at T0 (child's EBD total *T*‐score, mother's ADS score, father's ADS score), only the child's sex, age at diagnosis, and autism symptoms severity were associated with the child's EBD total *T*‐score at the *p* < 0.10 threshold after FDR correction (see Table S1). No covariates were associated with the mother's ADS score or the father's ADS score at T0. At T1, only the child's age at diagnosis was associated with one of the outcomes, the mother's ADS score at T1, after adjustment for the corresponding outcome at T0 (see Table S2). Consequently, for parsimonious CLPMs focusing on child–mother associations, the child's age at diagnosis was included as a covariate in the equations for both outcomes at T0 and T1. In contrast, for parsimonious CLPMs involving the father, no covariates were retained.

#### 3.2.1 CLPM Results Overview

The preregistered (theoretical), parsimonious, and baseline (without covariates) CLPMs were just identified (df = 0). As such, model fit could not be evaluated, and the results cannot be generalized beyond the sample (see Tables S5–S10). To address this limitation, we also estimated simplified baseline models in which the correlation between the variables of interest at T0 and/or the residual covariance between these variables at T1 were constrained to zero, as detailed in Supporting Information: Methods S2. These models, by introducing degrees of freedom, allowed evaluation of model fit. Furthermore, sensitivity analyses of the CLPMs (see Supporting Information: Methods S2), which showed acceptable to excellent fit (see Tables S11–S16), systematically adjusting for CBCL versions at T0 and T1, as well as child age at diagnosis, yielded congruent findings. These consistent results across analytical approaches strengthen the robustness of our conclusions. Consequently, in the manuscript we present only the results from the model showing the best fit indices.

### 3.3 Bidirectional Associations Between Mothers' ADS and Children's EBD at the Time of Autism Diagnosis and Three‐Year Follow‐Up

**FIGURE 3:**
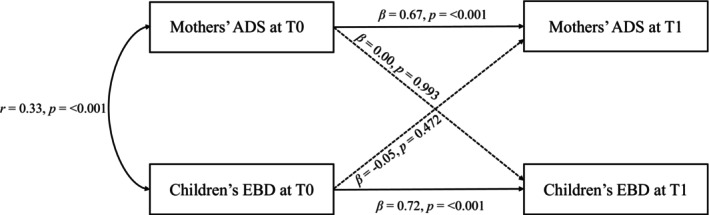
Cross‐Lagged panel models between mothers' anxiety/depressive symptoms (ADS) and emotional/behavioral difficulties (EBD) in autistic children following diagnosis in the mothers' analysis population (*n* = 315). The residual covariance at T1 was fixed to zero. Time points: T0, time of the child's autism diagnosis; T1, 3 years after T0.

The best‐fitting model to represent the bidirectional association between mother's ADS and children's EBD was the one without residual covariance between children's EBD and mothers' ADS at T1 (*χ* ^2^(1) = 2.61, *p* = 0.106, RMSEA = 0.12, CFI = 0.99, TLI = 0.93, SRMR = 0.02). This model showed an acceptable to excellent overall fit, with the exception of the RMSEA; however, such values are expected in models with very few degrees of freedom (Kenny et al. 2015). The CLPM results did not reveal a significant cross‐lagged effect between mothers' ADS at T0 and children's EBD at T1 (*β* = 0.00, *p* = 0.993), nor vice versa (*β* = −0.05, *p* = 0.472, see Figure 3 and Table S5). However, we found significant autoregressive coefficients for mothers' ADS (*β* = 0.67, *p* < 0.001) as well as for children's EBD (*β* = 0.72, *p* < 0.001), demonstrating the predictive influence of these variables on themselves over the 3 years following the child's autism diagnosis. A significant positive correlation was observed between mothers' ADS and children's EBD at T0 (*r* = 0.33, *p* < 0.001). Regarding the explained variance, the *R* ^2^ values were as follows: 0.52 for children's EBD at T1, and 0.43 for mothers' ADS at T1. These results suggest that the variances in children's EBD and mothers' ADS at T1 are largely explained by their respective measurements at T0, reflecting the strong stability of these variables. Similar conclusions were found in the mother's complete case population (*n* = 119, see Table S5), as well as across the other estimated models, although some fit could not be evaluated.

### 3.4 Bidirectional Associations Between Mothers' ADS and Two Subdimensions of Children's EBD: Internalizing and Externalizing Difficulties

**FIGURE 4:**
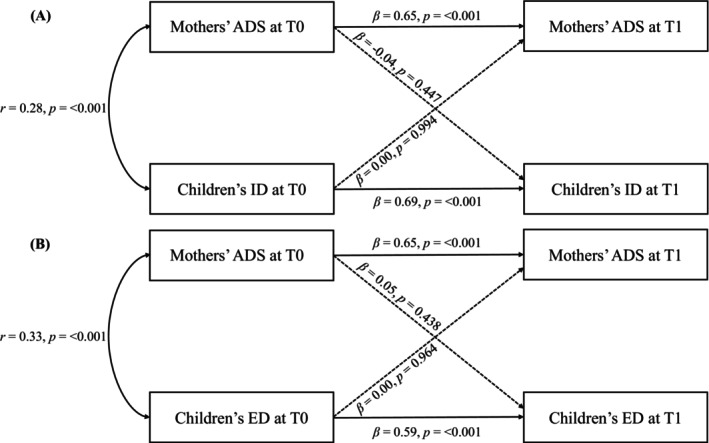
Cross‐lagged panel models between mothers' anxiety/depressive symptoms (ADS) and two subtypes of emotional/behavioral difficulties (EBD) in autistic children following diagnosis: (A) internalizing difficulties (ID) and (B) externalizing difficulties (ED) in the mothers' analysis population (*n* = 315). In both models, the residual covariance at T1 was fixed to zero. Time points: T0, time of the child's autism diagnosis; T1, 3 years after T0.

Regarding the bidirectional associations between mothers' ADS and children's internalizing difficulties (ID) or externalizing difficulties (ED), the best‐fitting specification was again the model without residual covariance at T1 (see Tables S6 and S7). The fit of this model was excellent for ID (*χ* ^2^(1) = 0.00, *p* = 0.982, RMSEA = 0.00, CFI = 1.00, TLI = 1.00, SRMR = 0.00) and similarly good for ED (*χ* ^2^(1) = 0.83, *p* = 0.362, RMSEA = 0.00, CFI = 1.00, TLI = 1.00, SRMR = 0.01). Analysis indicated nonsignificant cross‐lagged effects between mothers' ADS at T0 and children's ID at T1 (*β* = −0.04, *p* = 0.447), as well as vice versa (*β* = 0.00, *p* = 0.994, see Figure 4, Table S6). Likewise, no significant cross‐lagged effects were observed between mothers' ADS at T0 and children's ED at T1 (*β* = 0.05, *p* = 0.438), as well as vice versa (*β* = 0.00, *p* = 0.964, see Figure 4, Table S7). However, significant stability coefficients were found for the children's ID (*β* = 0.69, *p* < 0.001) and ED (*β* = 0.59, *p* < 0.001) between T0 and T1. A significant positive correlation was noted between mothers' ADS and children's ID at T0 (*r* = 0.28, *p* < 0.001) as well as between mothers' ADS and children's ED at T0 (*r* = 0.33, *p* < 0.001), indicating associations between these variables at T0. In these models, 46% of the variance in children's ID at T1 and 36% of the variance in children's ED at T1 were explained. Comparable results were observed in the mothers' complete case analysis of mothers (*n* = 119, see Tables S6 and S7).

### 3.5 Exploratory Analyses

#### 3.5.1 Bidirectional Associations Between Fathers' ADS and Children's EBD

**FIGURE 5:**
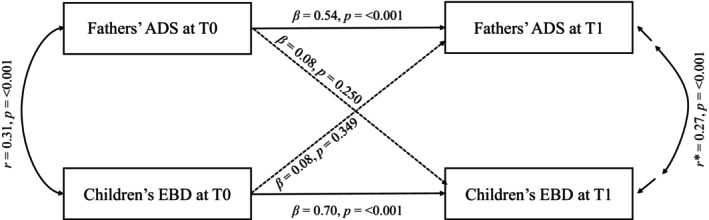
Cross‐lagged panel model between fathers' anxiety/depressive symptoms (ADS) and children's emotional/behavioral difficulties (EBD) in autistic children following diagnosis adjusted for CBCL version at T0 and T1, and child age at diagnosis (*n* = 213). Asterisk indicates residual that reflects the association between the residual variances of fathers' ADS and children's EBD at T1, after accounting for autoregressive effects, cross‐lagged effects and covariates (CBCL versions at T0 and T1, and child age diagnosis); Time points: T0, time of the child's autism diagnosis; T1, 3 years after T0.

The best‐fitting model to represent the bidirectional association between fathers' ADS and children's EBD was the base model (*n* = 213) adjusted on the CBCL versions at T0 and T1 as well as child age at diagnosis present in Table S14 (*χ* ^2^(6) = 19.46, *p* = 0.003, RMSEA = 0.11, CFI = 0.95, TLI = 0.84, SRMR = 0.07), showing an acceptable fit. We were unable to demonstrate significant cross‐lagged effects between fathers' ADS at T0 and children's EBD at T1 (*β* = 0.08, *p* = 0.250), and vice versa (*β* = 0.08, *p* = 0.349; see Figure 5 and Table S14). Moreover, significant stability coefficients were evident for fathers' ADS (*β* = 0.54, *p* < 0.001). The explained variance (*R* ^2^) values were 0.52 for children's EBD at T1 and 0.32 for fathers' ADS at T1. Significant positive correlations were found between fathers' ADS and children's EBD at T0 (*r* = 0.31, *p* < 0.001) and T1 (*r* = 0.27, *p* < 0.001).

#### 3.5.2 Bidirectional Associations Between Children's EBD and Mothers' Anxiety and Depressive Symptoms Studied Stratified

Regarding the bidirectional associations between children's EBD and mother's anxiety or depressive symptoms, the best‐fitting specification was again the model without residual covariance at T1 (see Tables S9 and S10). The fit of the model concerning mothers' anxiety was good to excellent, except for the RMSEA, which, as previously noted, is inflated due to the low degrees of freedom. In contrast, the model concerning mothers' depression showed an excellent overall fit. Like our previous findings, for both subdimensions of mothers' psychological distress, we did not detect any cross‐lagged effects (see Figure S2, Tables S9 and S10). Significant stability coefficients were observed for mothers' anxiety symptoms (*β* = 0.60, *p* < 0.001), as well as mothers' depressive symptoms (*β* = 0.68, *p* < 0.001) between T0 and T1. A significant correlation was observed between mothers' anxiety symptoms and children's EBD at T0 (*r* = 0.32, *p* < 0.001), as well as between mothers' depressive symptoms and children's EBD at T0 (*r* = 0.26, *p* < 0.001).

#### 3.5.3 Comparison of Parental Anxiety/Depressive Symptoms and Emotional/Behavioral Difficulties in Autistic Children Between Time of Child's Autism Diagnosis and 3 Years Later

Despite the significant strong autoregressive effects of EBD in children and ADS in parents, Wilcoxon tests reveal a decrease in symptoms from the time of the child's diagnosis to 3 years later (see Table 3). However, it is noteworthy that, according to clinical cutoffs, only the internalizing difficulties in children and the anxiety symptoms in fathers have displayed a clinically significant reduction over the three‐year period (see Table S17).

**TABLE 3:** Comparison of parental anxiety/depressive symptoms and emotional/behavioral difficulties in autistic children between the time of child's autism diagnosis and 3 years later in matched participants from the mothers' analysis population.

	*n*	Time of child's autism diagnosis	Three years after child's autism diagnosis	*p* *
		*M* (SD)	*M* (SD)	
Children's scores				
EBD total *T*‐score	175	65.15 (9.97)	63.58 (9.76)	0.004
Internalizing *T*‐score	175	64.91 (10.20)	62.67 (10.62)	< 0.001
Externalizing *T*‐score	177	60.28 (9.76)	58.13 (10.12)	0.001
Mothers' scores				
Total anxiety‐depression score	139	16.18 (6.61)	14.50 (7.54)	0.002
Anxiety score	142	9.53 (3.82)	8.80 (4.58)	0.046
Depression score	141	6.57 (3.59)	5.65 (3.95)	< 0.001
Fathers' scores				
Total anxiety‐depression score	80	13.89 (6.28)	11.81 (7.02)	0.013
Anxiety score	83	7.90 (3.39)	6.65 (3.84)	0.005
Depression score	82	5.95 (3.77)	5.12 (3.80)	0.056

Abbreviation: EBD, emotional and behavioral difficulties.

* Estimated using Wilcoxon tests.

## 4 Discussion

Using a two‐wave design, this study examined bidirectional associations between parental ADS and children's EBD at the time of the child's autism diagnosis (T0) and 3 years later (T1). In contrast with all previous studies, we did not observe any bidirectional or unidirectional association between parental ADS and children's EBD. However, our findings indicated moderate concurrent associations at T0 and strong autoregressive stability; at T1, a residual concurrent association was supported for fathers only (discussed below). Moreover, no bidirectional association was found when separately examining internalizing and externalizing difficulties nor when examining separately mothers' anxiety and depressive symptoms. Therefore, our results do not support the hypothesis of May and Williams (2024) suggesting that this bidirectional association would be observable in the years following the child's diagnosis. Similarly, we found no evidence of bidirectional associations between fathers' ADS and children's EBD. The consistency of our CLPM results across sensitivity analyses (see Tables S11–S16)—accounting for the CBCL version at T0 and T1 as well as child age at diagnosis—strengthens confidence in their robustness. Rather than revealing bidirectional or unidirectional effects, our findings emphasize two key patterns: (1) synchronous associations between parental ADS and children's EBD at diagnosis (*r* = 0.26 to *r* = 0.33 across models, *p* < 0.001), and (2) strong autoregressive effects, suggesting that early psychological distress in parents and children tends to persist over time, independent of mutual influence. The lack of evidence for cross‐lagged effects in our CLPMs may reflect the well‐documented time‐interval dependency of lagged parameters: with a three‐year lag and strong autoregressive stability, cross‐lagged effects can be attenuated and become difficult to detect (Voelkle et al. 2012). We therefore encourage future research—ideally with at least three measurement occasions—to use continuous‐time models, which can examine how cross‐lagged effects vary as a function of the measurement interval and help identify the time lag at which such effects may peak (Driver et al. 2025, 2017). Our results align with prior research confirming the well‐established synchronous association between parental ADS and children's EBD (May and Williams 2024; Piro‐Gambetti et al. 2023; Totsika et al. 2013; Yorke et al. 2018). These moderate associations likely reflect the substantial emotional burden experienced by parents during the initial phases of diagnosis and adjustment. However, at the 3‐year follow‐up (T1), the residual association between parental ADS and children's EBD remained significant only for fathers, as their best‐fitting model retained and supported this covariance. In contrast, for mothers, the best‐fitting model excluded the T1 covariance, meaning that its inclusion was not justified by the data, thus precluding any conclusion about a persistent association. This pattern may partly reflect stronger temporal stability in mothers' ADS than fathers' (*β* = 0.67 vs. *β* = 0.54), which may reduce residual variance at follow‐up and the detectability of a modest mother–child contemporaneous covariance at T1 (*β* = 0.15, *p* = 0.09). Another possibility is that mothers' symptoms become less tightly coupled with children's EBD over time as mothers gain experience, understanding, and confidence in managing their child's needs following diagnosis. Mothers, who more frequently assume primary caregiving, child's education, and organizational roles (Gray 2003; Hartley et al. 2014), may have had greater exposure to psychoeducational resources, therapeutic guidance, and peer support over time. These differences may help contextualize why the mother–child covariance was not retained at T1 in the best‐fitting model, without implying directional effects. An alternative, speculative explanation is that the fathers‐only covariance at T1 may reflect sex‐specific shared vulnerability that depends on the parent–child sex dyad. Given that 80% of our sample were boys and autism is diagnosed more often in boys than girls (Napolitano et al. 2022), a father–son pattern may have been more readily detectable in our data. Genetic or psychosocial vulnerabilities specific to father–son dyad could contribute to a persistent association between fathers' ADS and children's EBD across both time points. This hypothesis is exploratory and should be tested in future studies; however, our design does not allow us to directly examine sex‐specific pathways, and larger samples with greater representation of girls are needed to determine whether parent–child covariance patterns differ by child sex.

Inspecting *R* ^2^ values, at T0 the covariates (child's age, sex, IQ, autism symptoms severity, parental age, and education level) accounted for only a small proportion of variance in the key outcomes. At T1, *R* ^2^ from the preregistered just‐identified models was very similar to those from just‐identified baseline models without covariates, suggesting limited incremental explanatory power of the covariates in our dataset. These results should be interpreted cautiously given this study's limitations (sample size, two‐wave design, and the 3‐year lag between T0 and T1). Unmeasured factors, whether environmental or genetic, could simultaneously play an even more substantial role in the relationship between parental ADS and children's EBD. There are several environmental factors that are already known to influence parental ADS and/or children's EBD, such as social and familial support, parental stress levels, coping strategies, siblings or parent couple relationships, as well as traumatic events (Alibekova et al. 2022; Benson 2010; Hoover 2015; Liu et al. 2024; Piro‐Gambetti et al. 2023, 2024). Genetic factors have also been shown to be involved in the general population, between parents' ADS and children's EBD (Frach et al. 2024; Lewis and Plomin 2015; Silberg et al. 2010).

This research underlines the high prevalence of ADS among parents at the time of their child's autism diagnosis: 55.2% of mothers and 42.7% of fathers exhibited clinically significant ADS. At the time of diagnosis, 40.0% of mothers and 23.6% of fathers displayed clinically significant anxiety, while 16.5% of mothers and 11.0% of fathers suffered from clinically significant depression. These findings are consistent with Schnabel et al. (2020), who reported that 33% of parents of autistic children experienced anxiety. However, our study found a lower prevalence of clinically significant depression compared to the median of 31% (95% CI [0.24, 0.43]) observed by Schnabel et al. (2020) and the 22%–39% range reported by Piro‐Gambetti et al. (2023). Our anxiety prevalence also exceeded the prevalence reported in the French adult population in 2021 (13.00%; Léon et al. 2025), while depression prevalence was comparable (Léon et al. 2023). These differences may arise because HADS tends to yield lower depression rates than the Centre for Epidemiological Studies‐Depression Scale (Covic et al. 2009; Piro‐Gambetti et al. 2023). We recommend that future research aims to investigate concordance between depression measurement scales to better understand which one should be prioritized. In accordance with prior studies (Hastings et al. 2021; Totsika et al. 2013), we found high rates of co‐occurring EBD in autistic children, with 61.3% showing clinically significant symptoms at the time of diagnosis. Nevertheless, unlike Piro‐Gambetti et al. (2023), who revealed clinically significant internalizing difficulties in 26%–36%, we observed a higher prevalence amounting to 59.7%. This could be attributed to the timing of assessment, aligning with the child's diagnosis, a period of heightened distress for both children and parents (Bonis 2016; Rattaz et al., 2023; Vernhet et al. 2019). Moreover, our findings show that parental ADS and children's EBD at the time of autism diagnosis strongly predicted their values 3 years later, indicating symptom stability over time. However, we recorded significant improvements in parental ADS and children's EBD scores after 3 years although only reductions in children's internalizing difficulties and fathers' anxiety were clinically significant (see Table S17). While mothers also exhibited significant reductions in anxiety‐depressive symptoms over time, these improvements did not meet clinical significance thresholds. This divergence in clinical outcomes between mothers and fathers may reflect gender‐specific trajectories in mental health adjustment following an autism diagnosis, as well as differences in coping strategies. This finding aligns with literature showing that mothers often report higher levels of stress, anxiety and depressive symptoms (Dabrowska and Pisula 2010; Hastings et al. 2005; Jones et al. 2013; Rattaz et al., 2023), requiring larger absolute changes to achieve clinically meaningful improvements.

### 4.1 Study Strengths, Limitations, and Future Directions

Our study has several strengths. First, we used data from families enrolled at the time of their child's autism diagnosis, capturing a broad range of diagnostic timelines from early to later diagnoses. Diagnoses were systematically confirmed using DSM‐5 criteria through multidisciplinary assessments including at least the Autism Diagnosis Interview‐Revised (ADI‐R), the ADOS 2, a psychometric evaluation, and the Vineland Adaptive Behavior Scales (Vineland‐II). Second, we utilized mental health scales validated for our study populations, demonstrating good psychometric properties (Bocéréan and Dupret 2014; Pandolfi et al. 2009, 2012, 2014). The use of the CLPM framework further strengthens our study by allowing the simultaneous examination of temporal and bidirectional effects, providing a more comprehensive understanding of the dynamic relationship between parental ADS and children's EBD, thereby reducing bias from unidirectional analysis. Furthermore, we accounted for various covariates measured at the time of the child's diagnosis including age, sex, IQ, autism symptoms severity, and parental age and education level which are relevant to our variables of interest (Yorke et al. 2018), as well as CBCL versions. Finally, our study includes data on fathers and externalizing difficulties in autistic children, which are often missing in the literature.

Despite these strengths, there are notable limitations. The main limitation is the use of two different versions of the CBCL (CBCL/1.5–5 and CBCL/6–18) across measurement points, which raises the issue of heterotypic continuity: it is difficult to know whether observed changes reflect true developmental processes or result from version effects (Petersen et al. 2020). We mitigated this by using age‐normed *T*‐scores intended to be comparable across forms and by evaluating Petersen's six criteria; taken together, these analyses provided supportive evidence for temporal invariance of the constructs (see Supporting Information: Methods S1). We also conducted sensitivity analyses accounting for CBCL versions and child age at diagnosis as covariates. We could not apply more demanding approaches to assess measurement invariances such as the moderated nonlinear factor analysis (MNLFA; Bauer 2017) given sample‐size requirements, nor fit a longitudinal measurement model spanning both forms via anchor items (Kopf et al. 2015), which was not suitable here because overlapping items did not adequately cover the full constructs. For instance, the overlapping items for internalizing difficulties mainly reflect somatic complaints, which introduces bias and restricts construct coverage. Finally, we could not run a sensitivity analysis around the 5‐ to 6‐year form‐change window, as children entered at T0 between ages 2 and 16 and the T0–T1 interval was 3 years. Given the wide age range at diagnosis, age at diagnosis may moderate the associations between parental ADS and children's EBD. However, our two‐wave design and sample size did not allow for informative moderation analyses (e.g., age‐by‐ADS/EBD interactions or age‐stratified models). Future longitudinal work should examine age‐specific associations, particularly around school entry and adolescence. The study's reliance on only two measurement points restricts the application of more advanced statistical models, such as random‐intercepts‐CLPM (RI‐CLPM; Hamaker et al. 2015) or continuous‐time models (Driver et al. 2017). This limitation also restricts our ability to make inferences about the influence of time on the variables. Moreover, missing data at 3‐year follow‐up reduces the statistical power of our models although analyses of complete cases yielded similar results. The extended 3‐year interval between measurements may introduce temporal variations, confounding factors, and developmental changes that complicate the identification of bidirectional or unidirectional associations. Additionally, our sample underrepresent families of children with lower IQ, as these groups were more likely to be lost to follow‐up. Our findings may not generalize to families of children with intellectual disability. A key limitation is the substantial attrition from T0 to T1: retention was 62.2% for mothers (196/315) and 62.4% for fathers (133/213). This loss to follow‐up is likely attributable, at least in part, to the high caregiving burden and competing demands faced by parents of autistic children, the length of the questionnaire battery, and the absence of financial compensation for participation. Future studies should prioritize inclusive recruitment and retention strategies to ensure representation across the full autism spectrum. Lastly, the HADS used in our study may have been less sensitive in detecting depression compared to other scales (Piro‐Gambetti et al. 2023).

Future research should first replicate these findings in larger, more diverse samples to assess their generalizability. Additionally, studies using longitudinal data from large cohorts with at least three measurement points spaced no more than 2 years apart are needed to further investigate bidirectional associations, while incorporating additional family environmental factors. We recommend using continuous‐time models, which can separate within‐person change from stable between‐person differences and evaluate how associations vary as a function of the time lag between measurements (Voelkle et al. 2012; Driver et al. 2017). Further investigations are also needed to deepen our understanding of parental differences, particularly by examining whether these patterns vary across countries with distinct cultural norms and parental role expectations. Where sample size permits, researchers should also test longitudinal construct invariance of CBCL internalizing, externalizing, and total difficulties using MNLFA in the context of autism and ideally a common‐person bridge design (administering both CBCL versions to 5‐ to 6‐year‐olds) to minimize anchor bias. Clinicians should prioritize early screening and support for both parents and children, recognizing that their distress may persist independently. For example, interventions for parents to manage chronic stress and evidence‐based therapies for children (e.g., applied behavior analysis, cognitive‐behavioral therapy) could be implemented in parallel (Gitimoghaddam et al. 2022; Wang et al. 2021).

## 5 Conclusion

In summary, our research found no evidence of bidirectional or unidirectional associations between parental ADS and children's EBD 3 years after the child's autism diagnosis. Critically, we observed a high prevalence of parental ADS and EBD among autistic children at the time of diagnosis, with a strong association between these symptoms during this stressful period. However, the persistence of symptoms over time appears driven by autoregressive effects, not mutual influence. Future longitudinal studies should use continuous‐time models to examine whether parent–child associations are time‐lag dependent (including identifying potential peak lags) and to test whether these associations differ across parent–child sex dyads, particularly in samples with better representation of girls. Nevertheless, our findings underscore the need for systematic screening of both parental and child mental health at the time of diagnosis in France to identify families in need and provide timely support. They also highlight the importance of explicitly integrating parents' mental health into autism care pathways. More broadly, our results support the provision of concurrent—but not assumedly interdependent—support for parents and children, such as evidence‐based interventions targeting children's EBD alongside accessible mental health resources for parents, rather than approaches that presume improvements in one will necessarily translate into improvements in the other.

## Author Contributions

M.M. conducted the literature review and prepared the online preregistration. H.P. and A.B. reviewed the preregistration draft. M.P. and C.M. managed the data extraction. M.M. analyzed the data, drafted the initial manuscript, and drafted the initial responses to reviewers. M.M., H.P., Y.S., and C.M. participated in the selection of statistical analyses. H.P. provided weekly supervision to ensure the progress of the project. A.B. is the Principal Investigator of the ELENA cohort. All Authors contributed to data interpretation, critically revised the research content, and approved the final manuscript.

## Funding

This research was supported by grants from the French Health Ministry (DGOS) (PHRCN 2013, Grant 13‐0232) and the National Solidarity Fund for Autonomy (Grant 030319). The Montpellier University Hospital (AOI) provided additional support. M.M. received a doctoral scholarship from the University of Versailles Saint‐Quentin‐in‐Yvelines to conduct this research as part of her PhD thesis.

## Ethics Statement

The study and informed consent procedure were approved by the Marseille Mediterranean Ethics Committee and the French Data Protection Agency (CNIL, authorization number DR‐2015‐393).

## Consent

All participating families signed an informed consent form.

## Conflicts of Interest

The authors declare no conflicts of interest.

## Supporting information


**Data S1:** aur70220‐sup‐0001‐Supinfo.docx.

## Data Availability

Data are available upon reasonable request and subject to approval by A.B., the Principal Investigator. Portions of the code that support the findings of this study are available from the corresponding author, M.M., upon reasonable request. M.M. confirms that she had full access to all the data in the study and takes responsibility for the integrity of the data and accuracy of the data analysis.
