# Supplementary material for: Reciprocal Associations Between Parental Anxiety/Depression and Emotional/Behavioral Difficulties in Autistic Children Following Their Diagnosis
Source: Autism Res. 2026 Mar 19;19(4):e70220. doi: 10.1002/aur.70220 (PMC13087833; doi:10.1002/aur.70220)
Supplement: Supplementary file 1 — Data S1: aur70220‐sup‐0001‐Supinfo.docx. [file AUR-19-0-s001.docx]

**List of Supporting Information**

**Supp. Fig. 1.** Distribution of parental Anxiety/Depression scores and Emotional/Behavioral Difficulties in autistic children across time points: (T0) Time of child’s autism diagnosis and (T1) Three years after T0.

**Supp. Fig. 2.** CLPM between children’s Emotional/Behavioral Difficulties (EBD) and **(A)** mothers’ Anxiety Symptoms (AS) as well as **(B)** mothers’ Depression Symptoms (DS) following the child’s autism diagnosis in the mothers’ analysis population (*n* = 315).

**Supp. IQ Score Algorithm**

**Supp. Methods S1.** Evaluating construct validity invariance of Internalizing, Externalizing and Total difficulties across CBCL versions (1½-5 & 6-18) using Petersen et al.’s (2020) six-criteria.

**Supp.** **Methods S2.** Additional methodological details, model comparison in Cross-Lagged Panel Modeling, and interpretation of CLPM adequacy.

**Supp. Handling of missing data**

**Supp. Table 1.** Univariate analyses of associations between covariates and the main CLPM outcome scores at baseline (T0).

**Supp. Table 2.** Associations between baseline covariates and outcomes at T1 (linear regression models adjusted for baseline outcome).

**Supp. Table 3.** Comparison of family characteristics at the time of child's autism diagnosis between the mothers’ analysis population (*n* = 315) and those who were excluded (*n* = 561) from the ELENA cohort.

**Supp. Table 4.** Comparison of family characteristics at the time of child's autism diagnosis between the mothers’ complete cases population (*n* = 119) and those who were excluded (*n* = 196) from mothers’ analysis population.

**Supp. Table 5.** Standardized estimates and assessment of Cross-Lagged Panel Models' adequacy for the bidirectional association between mothers' Anxiety/Depression Symptoms and Emotional/Behavioral Difficulties in autistic children following diagnosis.

**Supp. Table 6.** Standardized estimates and assessment of Cross-Lagged Panel Models' adequacy for the bidirectional association between mothers' Anxiety/Depression Symptoms and Internalizing Difficulties in autistic children following diagnosis.

**Supp. Table 7.** Standardized estimates and assessment of Cross-Lagged Panel Models’ adequacy for the bidirectional association between mothers' Anxiety/Depression Symptoms and Externalizing Difficulties in autistic children following diagnosis.

**Supp. Table 8.** Standardized estimates and assessment of Cross-Lagged Panel Models' adequacy for the bidirectional association between fathers’ Anxiety/Depression Symptoms and Emotional/Behavioral Difficulties in autistic children following diagnosis (*n* = 213).

**Supp. Table 9.** Standardized estimates and assessment of Cross-Lagged Panel Models' adequacy for the bidirectional association between mothers’ Anxiety Symptoms and Emotional/Behavioral Difficulties in autistic children following diagnosis (*n* = 315).

**Supp. Table 10.** Standardized estimates and assessment of Cross-Lagged Panel Models' adequacy for the bidirectional association between mothers’ Depression Symptoms and Emotional/Behavioral Difficulties in autistic children following diagnosis (*n* = 315).

**Supp. Table 11.** Sensitivity analysis of CLPM: Bidirectional association between mothers' Anxiety/Depression Symptoms and Emotional/Behavioral Difficulties in autistic children following diagnosis adjusted for CBCL version at T0 and T1, and child age at diagnosis (*n* = 315).

**Supp. Table 12.** Sensitivity analysis of CLPM: Bidirectional association between mothers' Anxiety/Depression Symptoms and Internalizing Difficulties in autistic children following diagnosis adjusted for CBCL version at T0 and T1, and child age at diagnosis (*n* = 315).

**Supp. Table 13.** Sensitivity analysis of CLPM: Bidirectional association between mothers' Anxiety/Depression Symptoms and Externalizing Difficulties in autistic children following diagnosis adjusted for CBCL version at T0 and T1, and child age at diagnosis (*n* = 315).

**Supp. Table 14.** Sensitivity analysis of CLPM: Bidirectional association between fathers’ Anxiety/Depression Symptoms and Emotional/Behavioral Difficulties in autistic children following diagnosis adjusted for CBCL version at T0 and T1, and child age at diagnosis (*n* = 213).

**Supp. Table 15.** Sensitivity analysis of CLPM: Bidirectional association between mothers’ Anxiety Symptoms and Emotional/Behavioral Difficulties in autistic children following diagnosis adjusted for CBCL version at T0 and T1, and child age at diagnosis (*n* = 315).

**Supp. Table 16.** Sensitivity analysis of CLPM: Bidirectional association between Depression Symptoms and Emotional/Behavioral Difficulties in autistic children following diagnosis adjusted for CBCL version at T0 and T1, and child age at diagnosis (*n* = 315).

**Supp. Table 17.** Clinical comparison of parental Anxiety/Depression Symptoms and Emotional/Behavioral Difficulties in autistic children between time of children's autism diagnosis and three years later in matched participants from the mothers’ analysis population.


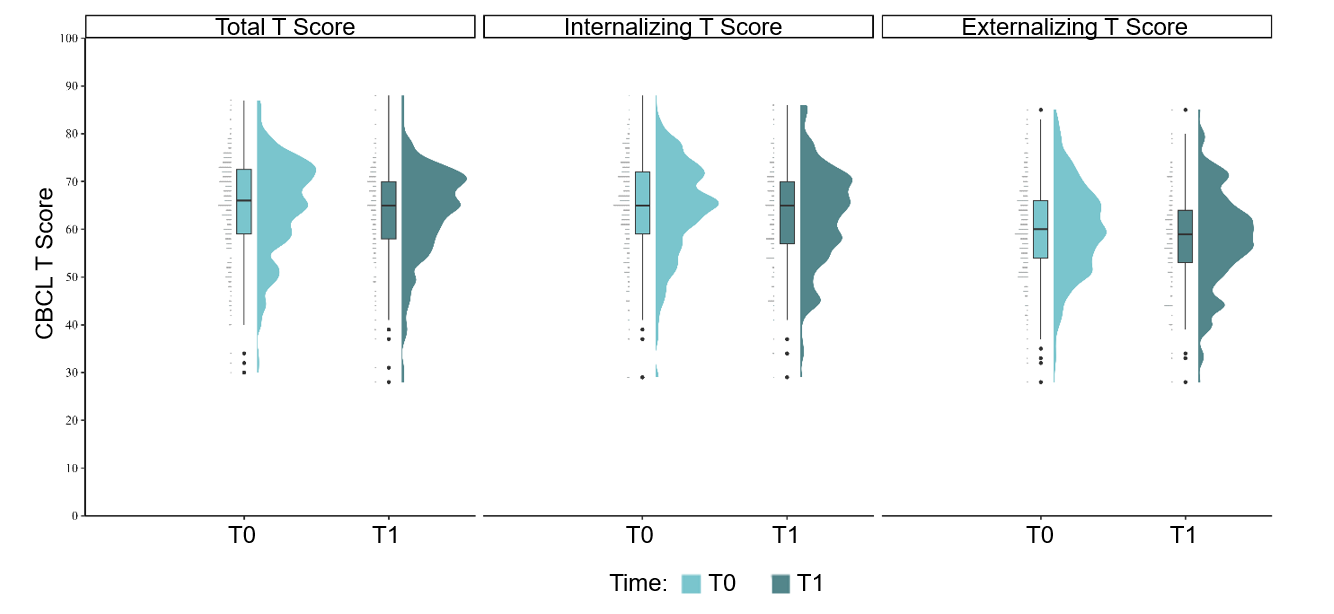

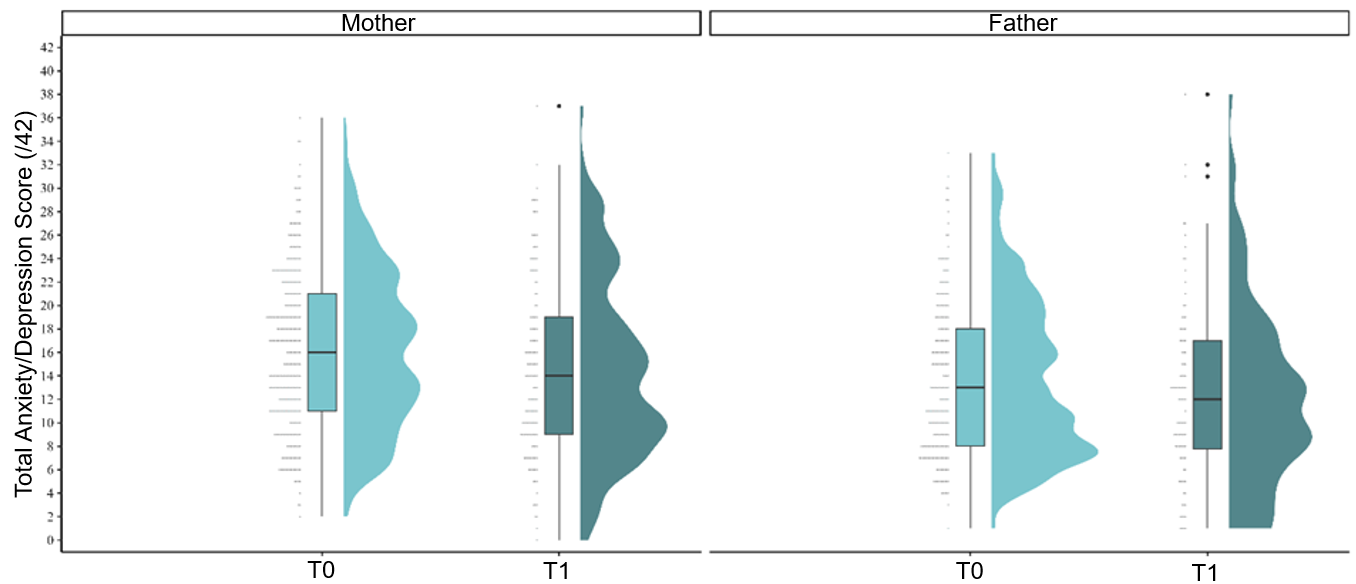


**Supp. Fig. 1.** Distribution of parental Anxiety/Depression scores and Emotional/Behavioral Difficulties in autistic children across time points: (T0) Time of child’s autism diagnosis and (T1) Three years after T0.

**(B)**

**(A)**


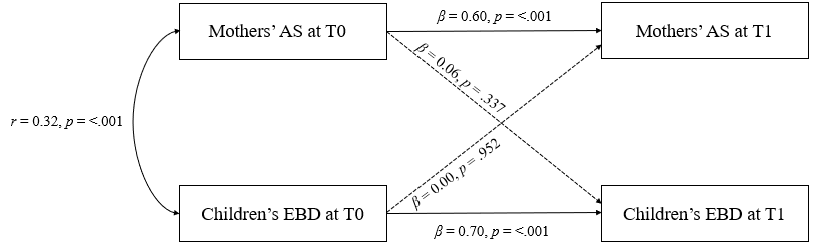


**(A)**

**(B)**


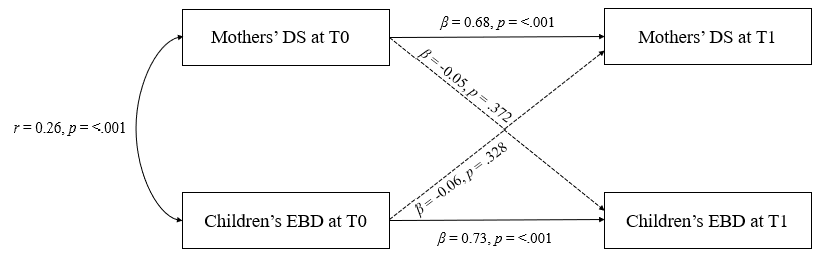


**Supp. Fig. 2.** CLPM between children’s Emotional/Behavioral Difficulties (EBD) and **(A)** mothers’ Anxiety Symptoms (AS) as well as **(B)** mothers’ Depression Symptoms (DS) following the child’s autism diagnosis in the mothers’ analysis population (*n* = 315).

*Note.* In both models, the residual covariance at T1 was fixed to zero. Time points: T0: time of the child’s autism diagnosis; T1: three years after T0.

**Supp. IQ Score Algorithm**

The IQ score algorithm estimates are derived from the “fluid reasoning” dimension of the WISC-V (The Wechsler Intelligence Scale for Children, Fifth Edition) (Wechsler, 2014a) and the WPPSI-IV (The Wechsler Preschool and Primary Scale of Intelligence, Fourth Edition) (Wechsler, 2014b), the “perceptual reasoning” dimension of the WISC-IV (Wechsler, 2003), and the “performance IQ” dimension of the WPPSI-III [(Wechsler, 2004)](https://www.zotero.org/google-docs/?Wch1RO) or “simultaneous processing” dimension of the Kaufman Assessment Battery for Children (KABC-II) (Kaufman & Kaufman, 2004) depending on the child's age. IQ-score < 40 is interpreted as profound disability, score *∈* [40 ; 55[ as moderate disability, score *∈* [55 ; 70[ as mild disability, and score ≥ 70 as no disability. These terms are used to align with established IQ classifications in clinical and research settings. However, we acknowledge that such labels do not reflect the individual strengths, abilities, or support needs of autistic individuals and may not align with contemporary inclusive language guidelines.

Kaufman, A. S., & Kaufman, N. L. (2004). *Kaufman Assessment Battery for Children Second Edition*. American Guidance Service.

Wechsler, D. (2003). *Wechsler Intelligence Scale for Children—Fourth Edition* (The Psychological Corporation).

Wechsler, D. (2014a). *WISC-V : Administration and Scoring Manual*. PsychCorp.

Wechsler, D. (2014b). *WPPSI-IV : Echelle d’intelligence de Wechsler pour enfants—Manuel d’interprétation* (4ème édition). ECPA.

**Supp.** **Methods S1.** Evaluating construct validity invariance of Internalizing, Externalizing and Total Difficulties across CBCL versions (1½-5 & 6-18) using Petersen et al.’s (2020) six-criteria.

1. **Content validity**

Internalizing, externalizing and total difficulties are constructs whose manifestations change with age (*heterotypic continuity*). This developmental shift in symptom expression motivates the use of two CBCL versions (1½-5 and 6-18). Internalizing difficulties reflects difficulties within the self (e.g., anxiety/depression, withdrawal, somatic complaints), whereas externalizing difficulties reflects conflicts with others (e.g., rule-breaking, aggression). Total difficulties regroups internalizing difficulties, externalizing difficulties and others emotional/behavioral difficulties. As shown in the table below, both versions cover the same broad domains: Internalizing, Externalizing, and Total difficulties, reflecting a common conceptual core with age-appropriate adaptations. In the CBCL/1½-5, for example, Emotionally reactive and Sleep difficulties are included, and Attention difficulties is grouped under Externalizing difficulties. In the CBCL/6-18, Rule-breaking behavior, Social difficulties, and Thought difficulties appear, and Attention difficulties becomes a distinct syndrome while still contributing to Total difficulties.

Item selection for the CBCL was based on “extensive literature searches, consultation with mental health professionals and special educators, and pilot testing with parents, youths, and teachers” (Achenbach & Rescorla, 2001, p. 108). Moreover, the content validity of CBCL “has been strongly supported by nearly four decades of research, consultation, feedback, and refinement, as well as by the current evidence for the ability of all items to discriminate significantly between demographically similar referred and nonreferred children” (Achenbach & Rescorla, 2001, p. 109).

|  | CBCL/1½-5 | CBCL/6-18 |
| --- | --- | --- |
| Internalizing difficulties | Emotionally reactive (9 items)  Anxious/depressed (8 items)  Somatic complaints (11 items)  Withdrawn (8 items) | Anxious/depressed (13 items)  Withdrawn/depressed (8 items)  Somatic complaints (11 items) |
| Externalizing difficulties | Attention difficulties (5 items)  Aggressive behavior (19 items) | Rule-breaking behavior (17 items)  Aggressive behavior (18 items) |
| Total difficulties | Internalizing difficulties (36 items)  Externalizing difficulties (24 items)  Sleep difficulties (7 items)  Other difficulties (33 items) | Internalizing difficulties (32 items)  Externalizing difficulties (35 items)  Social difficulties (11 items)  Thought difficulties (15 items)  Attention difficulties (10 items)  Other difficulties (17 items) |

1. **Test-retest reliability**

We could not estimate **test-retest reliability** in our study because approximately three years elapsed between assessments. However, **rank-order stability** were reported using Pearson correlations over an 8-day interval by Achenbach and Rescorla (2000, 2001). For **CBCL/1½-5** (*n* = 78), stability was **excellent** for Internalizing difficulties (r = .90) and Total difficulties (r = .90), and **good** for Externalizing difficulties (r = .87) (Achenbach & Rescorla, 2000). For **CBCL/6-18** (*n* = 73), stability was **excellent** across all three domains: Internalizing (r = .91), Externalizing (r = .92), and Total difficulties (r = .94) over the same 8-day interval (Achenbach & Rescorla, 2001).

1. **Convergent validity and discriminant validity**

**CBCL/1½-5**

Convergent validity between CBCL/1½-5 and Strengths and Difficulties Questionnaire (SDQ), both measure emotional and behavioral difficulties in children and adolescents, was good at age 2 in a U.S. sample (*n* = 132): Internalizing *r* = .54, Externalizing *r* = .58, and Total difficulties r = .55, all *p* < .001 (Byrne et al., 2024). In addition, **Achenbach & Rescorla (2000)** reported evidence of convergent validity for the **CBCL/2-3** with the **Richman Behavior Checklist** and the **Toddler Behavior Screening Inventory** (Total difficulties), as well as convergent associations between CBCL/2–3 Internalizing/Externalizing and the **Infant-Toddler Social and Emotional Assessment (ITSEA)**. Because only **two items** differ between the **CBCL/1½-5** and the **CBCL/2-3** (across 100 items), the authors noted that **similar convergence is expected** for the CBCL/1½-5. Consistent with divergent validity, **Achenbach & Rescorla (2000)** also reported **small** correlations between the **CBCL/2-3 Total** difficulties score and the **Bayley**, **McCarthy**, or **MCDI** scores (n = 86), as anticipated given their different constructs.

**CBCL/6-18**

Raw scores for **Internalizing**, **Externalizing**, and **Total difficulties** on the CBCL/6-18 showed very strong correlations with the corresponding CBCL-1991 scales (*r* = .98, .99, and 1.00, respectively; Achenbach & Rescorla, 2001). In turn, the CBCL-1991 broadband scales correlated strongly with conceptually matched **SDQ** domains: **Emotional symptoms**, **Conduct difficulties**, and **Total difficulties** (r = .74, .84, .87; all *p* < .001; Goodman & Scott, 1999).

**In the context of autism,** Pandolfi et al. (2014) reported **moderate-to-large, statistically significant** correlations between **CBCL/6-18 Internalizing difficulties** and **K-SADS Anxiety** (r = .65, p < .001) and **K-SADS Depression** (r = .66, p < .001), supporting the **convergent validity** of the Internalizing difficulties construct. They also found a **small, non-significant** association with the **ADI-R** (r = .14, p > .05), consistent with **discriminant validity**. **By contrast,** the **CBCL Total difficulties** scale correlated significantly with **K-SADS Anxiety**, **K-SADS Depression**, and the **ADI-R**, as expected given that Total difficulties aggregates both **internalizing** and **externalizing** difficulties.

We were not able to evaluate convergent validity in the present study (no concurrent external validators were available at baseline). However, in the study sample (*n*= 315) at T0, **173** children completed the **CBCL/1½-5** and **142** completed the **CBCL/6-18**. Using the IQ score defined in the **Supp. IQ Score Algorithm**, correlations between **CBCL broadband *T*-scores** and **IQ** were small and non-significant. CBCL/1½-5 (n = 173): Internalizing-IQ, r = −.02, p = .777; Externalizing–IQ, r = −.02, p = .759; Total difficulties–IQ, r = −.04, p = .579. CBCL/6–18 (n = 142): Internalizing-IQ, r = .13, p = .110; Externalizing-IQ, r = −.05, p = .518; Total difficulties–IQ, r = .03, p = .716. These findings are consistent with **discriminant validity**, given the conceptual distinctness of behavioral/emotional difficulties and cognitive ability.

Together, these findings provide robust evidence of convergent validity and discriminant validity for the broadband constructs (Internalizing difficulties, Externalizing difficulties and Total difficulties) of both CBCL versions.

1. **Similar factor structure**

Structural validity (CBCL/1½-5):

In an autism context, a U.S. study by Pandolfi et al. (2009) made it possible to estimate a confirmatory factor analysis with correlated Internalizing and Externalizing factors using syndrome scores (rather than items), due to the sample size (*n* = 128). The results support the two-factor model with acceptable fit. All factor loadings are significant, with moderate to large effect sizes. The correlation between Internalizing and Externalizing is high (*r* = .73, *p* < .05), supporting the possibility of a higher-order factor (Total difficulties) underlying the two domains.

Structural validity (CBCL/6-18):

Using a methodology similar to their 2009 study, **Pandolfi et al. (2012)** supported a **two-factor model** (Internalizing, Externalizing). Global fit was acceptable, with **CFI** and **SRMR** indicating good fit. All syndrome loadings were significant: **.59-.91** on Internalizing difficulties and **.65-.97** on Externalizing difficulties. The factors were moderately correlated (***r* = .59, *p* < .05**). By contrast, a **one-factor** solution showed inadequate fit. Although the factors share only about one-third of their variance, they retain distinctiveness, a pattern that aligns with the presence of a higher-order Total difficulties factor (Pandolfi et al., 2012).

1. **High internal consistency**

**Internal consistency** for the **Internalizing**, **Externalizing**, and **Total** difficulties scales of the CBCL/1½-5 and CBCL/6-18 versions is **good to excellent**, as indicated by findings from the ELENA cohort in France (*n =* 212 for CBCL/1½-5 and *n* = 176 for CBCL/6-18), Pandolfi et al. (2009, 2012), and the Achenbach & Rescorla manuals (2000, 2001).

In these supplementary analyses, conducted to evaluate the CBCL’s internal consistency in a French autism context, we drew subsamples from the ELENA cohort. Among the 876 autistic children at baseline, we included **212** children who completed all **CBCL/1½-5** items at baseline (optional items excluded from the completeness criterion) and **176** children who completed all **CBCL/6–18** items (optional items likewise excluded). We report **Cronbach’s α** and **McDonald’s ω**.

See the respective **Cronbach’s alpha** and Guttman’s λ_2_ coefficients below, as well as **McDonald’s omega** values observed in our cohort.

**Table 1.** Internal Consistency (Cronbach’s α) of CBCL/1½-5: Internalizing, Externalizing, and Total difficulties

|  | Our study (France; *n* = 212 autistic children) | Pandolfi et al., 2009, Table 3  (U.S., *n* = 128 autistic children) | Achenbach & Rescorla, 2000, p.155  (U.S., *n* = 1126; 563 referred; 563 nonreferred)  USA |
| --- | --- | --- | --- |
| Internalizing difficulties | .87 | .80 | .89 |
| Externalizing difficulties | .89 | .90 | .92 |
| Total difficulties | .94 | .93 | .95 |

*Note.* In our study*,* for the **CBCL/1½-5**, optional items **100a, 100b, and 100c** were not included in internal consistency estimates.

**Table 2.** Internal Consistency (Cronbach’s α or Guttman’s λ_2_) of CBCL/6-18: Internalizing, Externalizing, and Total difficulties

|  | Our study (France, *n* = 176 autistic children)  Cronbach’s α | Pandolfi et al., 2012  (U.S., *n* = 122 autistic children)  Guttman’s λ_2_ | Achenbach & Rescorla, 2001  (U.S., *n* = 3210; 1605 referred;1605 nonreferred)  Cronbach’s α |
| --- | --- | --- | --- |
| Internalizing difficulties | .88 | .90 | .90 |
| Externalizing difficulties | .87 | .90 | .94 |
| Total difficulties | .94 | .94 | .97 |

*Note.* In our study, for the **CBCL/6-18**, optional items **56h** and **113a, 113b, and 113c** were not included; item **105** was also excluded because it showed **zero variance** in our cohort.

**Table 3.** McDonald’s Omega (ω) for CBCL/1½-5 & CBCL/6-18: Internalizing, Externalizing, and Total difficulties in the ELENA French cohort.

|  | **CBCL/1½-5**  Our study  (France, *n* = 212 autistic children) | **CBCL/6-18**  Our study  (France, *n* = 176 autistic children) |
| --- | --- | --- |
| Internalizing difficulties | .88 | .88 |
| Externalizing difficulties | .89 | .88 |
| Total difficulties | .94 | .94 |

*Note.* For the **CBCL/1½-5**, optional items **100a, 100b, and 100c** were not included in internal consistency estimates. For the **CBCL/6-18**, optional items **56h** and **113a, 113b, and 113c** were not included; item **105** was also excluded because it showed **zero variance** in our cohort.

**6. Sensitive to change and show theoretically change across development**

**CBCL/1½-5**

To evaluate sensitivity to change in CBCL/1½–5 *T*-scores (Internalizing, Externalizing, Total difficulties), we ran paired *t*-tests on Δ = T1 - T0 (negative = improvement), and individual-level Reliable Change Index (RCI) using test–retest reliability (.90 for Internalizing and Total difficulties; .87 for Externalizing difficulties) from Achenbach & Rescorla (2000), and *T* score SD = 10. Reliable improvement was defined as **RCI ≤ -1.96**, reliable deterioration as **RCI ≥ +1.96**, and values in between as stable. Analyses were restricted to the 18 autistic children (within the mothers’ analytic sample, *n* = 315) who completed the same CBCL/1½-5 version at T0 and T1. **Using a bidirectional criterion of responsiveness,** we summarized the proportion with any reliable change (|RCI| ≥ 1.96), regardless of direction, and interpreted this “non-stable” rate against the 5% chance level implied by a 95% RCI

Over 3 years in the CBCL/1½-5 subsample (n=18), we found the following results:

- **Internalizing** difficulties, t(17) = 0.59, p = .562, Mdiff = 1.11 T, 95% CI [-2.85, 5.07], **RCI**: 5.6% improved / 77.8% stable / 16.7% deteriorated;
- **Externalizing** difficulties, t(17) = 0.85, p = .407, Mdiff = 1.78 T, 95% CI [-2.63, 6.18], **RCI**: 11.1% / 72.2% / 16.7%;
- **Total** difficulties, t(17) = 0.50, p = .624, Mdiff = 0.94 T, 95% CI [-3.05, 4.94], **RCI**: 11.1% / 66.7% / 22.2%.

**CBCL/6-18**

Across CBCL/1½-5 domains, paired t tests were non-significant, but the proportion with reliable change was **22.2%** (Internalizing), **27.8%** (Externalizing), and **33.3%** (Total difficulties) showed reliable change, well above the 5% chance rate, **indicating bidirectional sensitivity to change in either direction** in this n = 18 subsample, with shifts skewed toward deterioration.

**CBCL/6-18**

To evaluate sensitivity to change in CBCL/6-18 *T*-scores (Internalizing, Externalizing, Total difficulties) in the ELENA cohort, we applied the same procedure as for CBCL/1½-5 among the 84 autistic children who completed CBCL/6-18 at both waves (T0 and T1). Results were as follows:

- **Internalizing** difficulties: t(83) = -1.53, p = .130, Mdiff = -1.17 T, 95% CI [-2.68, 0.35], **RCI**: 10.7% improved / 81.0% stable / 8.3% deteriorated;
- **Externalizing** difficulties: t(83) = -4.60, p = <.001, Mdiff = -4.06 T, 95% CI [-5.82, -2.30], **RCI**: 33.3% / 61.9% / 4.8%;
- **Total** difficulties: t(83) = -3.78, p = <.001, Mdiff = -2.63 T, 95% CI [-4.02, -1.25], **RCI**: 25.0% / 66.9% / 7.1%.

In the CBCL/6-18 subsample (n = 84), paired t tests indicated significant mean change for Externalizing and Total difficulties but not for Internalizing difficulties. Using a bidirectional criterion (|RCI| ≥ 1.96), reliable change occurred in 19.0% (Internalizing), 38.1% (Externalizing), and 33.1% (Total), supporting sensitivity to change with a predominance of improvement.

Despite being age-standardized, *T*-scores (Internalizing, Externalizing and Total difficulties) from both the CBCL/1½-5 and CBCL/6-18 versions demonstrated sensitivity to change at the individual level for both versions. Additionally, the CBCL/6-18 *T*-scores also showed sensitivity to group-level change over the 3-year period. It is possible that similar group-level changes would be observed in the CBCL/1½-5 with a larger sample size, given that our subsample was limited to 18 autistic children compared to 84 autistic children in the CBCL/6-18 group.

Achenbach, T. M., & Rescorla, L. A. (2000). *Manual for the ASEBA preschool forms & profiles*. University of Vermont, Research Center for Children, Youth & Families.

Achenbach, T. M., & Rescorla, L. A. (2001). *Manual for the ASEBA School-Age Forms & Profiles*. University of Vermont, Research Center for Children, Youth & Families.

Byrne, E. M., Eneberi, A., Barker, B., Grimas, E., Iles, J., Pote, H., Ramchandani, P. G., & O’Farrelly, C. M. (2024). Psychometric properties of the preschool strengths and difficulties questionnaire (SDQ) in UK 1-to-2-year-olds. *European Journal of Pediatrics*, *183*(12), 5339–5350. https://doi.org/10.1007/s00431-024-05801-2

Goodman, R., & Scott, S. (1999). Comparing the Strengths and Difficulties Questionnaire and the Child Behavior Checklist: Is Small Beautiful? *Journal of Abnormal Child Psychology*, *27*(1), 17–24. https://doi.org/10.1023/A:1022658222914

Pandolfi, V., Magyar, C. I., & Dill, C. A. (2009). Confirmatory factor analysis of the child behavior checklist 1.5-5 in a sample of children with autism spectrum disorders. *Journal of Autism and Developmental Disorders*, *39*(7), 986–995. https://doi.org/10.1007/s10803-009-0716-5

Pandolfi, V., Magyar, C. I., & Dill, C. A. (2012). An initial psychometric evaluation of the CBCL 6–18 in a sample of youth with autism spectrum disorders. *Research in Autism Spectrum Disorders*, *6*(1), 96–108. https://doi.org/10.1016/j.rasd.2011.03.009

Pandolfi, V., Magyar, C. I., & Norris, M. (2014). Validity Study of the CBCL 6–18 for the Assessment of Emotional Problems in Youth With ASD. *Journal of Mental Health Research in Intellectual Disabilities*, *7*(4), 306–322. https://doi.org/10.1080/19315864.2014.930547

Petersen, I. T., Choe, D. E., & LeBeau, B. (2020). Studying a Moving Target in Development: The Challenge and Opportunity of Heterotypic Continuity. *Developmental Review: DR*, *58*, 100935. https://doi.org/10.1016/j.dr.2020.100935

**Supp.** **Methods S2.** Additional methodological details, model comparison in Cross-Lagged Panel Modeling, and interpretation of CLPM adequacy.

Before estimating the Cross-Lagged Panel Models (CLPMs), we conducted univariate analyses to identify covariates associated with the outcomes at baseline (T0). These analyses, reported in **Supp. Table 1**, used appropriate statistical tests based on the nature of the covariates: the Mann-Whitney U test for binary covariates, Pearson correlation for continuous covariates with linear relationships, Spearman correlation for monotonic but non-linear relationships, and distance correlation (dCor) when associations were neither linear nor monotonic. A CLPM is a type of structural equation model used to examine the directional relationships between variables over time with at least two time points. It estimates multiple parameters simultaneously, including autoregressive effects (the influence of a variable on itself later), cross-lagged effects (the influence of one variable on another variable later), and synchronous effects (the relationship between variables measured at the same time point). It helps identify whether alterations in one variable predict changes in another variable at later time points and provide some information about potential causal influences in longitudinal data. We employed Robust Maximum Likelihood estimation for parameter estimation in the CLPM models, due to its resilience to violations of homoscedasticity and the normality of residuals, as well as its robustness to outliers or extreme values. To assess the adequacy of our preregistered models and detect potential overfitting, we estimated several simplified models. These models (see **Supp. Table 5** to **Supp. Table 11**) include: (1) a parsimonious covariate-adjusted model, (2) a base model without adjustment variables, (3) a model without covariance between my variables of interest at T1, (4) a model without covariance between my variables of interest at T0, (5) a model without covariance between my variables at T0 and T1, plus (6) a sensitivity model adjusted only for CBCL version (T0/T1) and age at diagnosis. For model (1), parsimonious empirical CLPMs were estimated by retaining only covariates that prospectively predicted the T1 outcome while controlling for the corresponding T0 score (see **Supp. Table 2**). To address multiplicity, *p*-values were adjusted using the False Discovery Rate (FDR); associations were considered significant at *p* < .10**.** Model 6 was included to assess the stability of autoregressive, cross-lagged, and synchronous effects while controlling for potential biases related to differences in CBCL versions (see **Supp. Table 11** to **Supp. Table 16**).

The comparison between these simplified models and our preregistered models was conducted using the Sample-Size Adjusted Bayesian Information Criterion (SABIC), favoring the model with the lowest SABIC, and therefore indicating better model fit. The SABIC is used to compare different models and helps select the one that offers the best balance between model fit and complexity, considering the sample size (Hooper et al., 2008). According to the guidelines of Hooper et al. (2008), the fit of CLPM were also evaluated using several indices: the Chi square statistic (*nb.,* sensitive to sample size), the Root Mean Square Error of Approximation (RMSEA), the Tucker-Lewis Index (TLI), the Comparative Fit Index (CFI), and the Standardized Root Mean Square Residual (SRMR). A non-significant p-value associated with the Chi-square suggests a good fit of the model.

An RMSEA of ≤ .05 indicates an excellent fit, an RMSEA between .05 and .08 corresponds to an acceptable fit, values between .08 and .10 as a mediocre fit, while values > .10 are not acceptable. TLI and CFI values of ≥ .95 indicate an excellent fit, and if these values fall between .90 and .95, they correspond to an acceptable fit. Finally, an SRMR of ≤ .08 suggests a good fit. Due to the violation of certain assumptions such as homoscedasticity and/or normality of residuals in the CLPM and in the regression linear models, robust estimators accommodating these violations were employed.

Hamaker, E. L., Kuiper, R. M., & Grasman, R. P. P. P. (2015). A critique of the cross-lagged panel model. *Psychological Methods*, *20*(1), 102‑116. https://doi.org/10.1037/a0038889

Hooper, D., Coughlan, J., & Mullen, M. (2008). *Structural Equation Modelling : Guidelines for Determining Model Fit*. *6*(1).

**Supp. Handl****ing of missing data**

We evaluated missing data mechanisms via logistic regressions, employing the *glm()* function to ascertain Missing At Random (MAR) patterns. Given associations between some of our missing variables and the observed ones, we assumed that the data are MAR. Given the proportion of missing data on covariates (2-7%) and the substantial proportion of missing data on the variables of interest measured 3 years later (44-62%), Full Information Maximum Likelihood (FIML) was preferred over the combined MICE + FIML approach (Enders & Bandalos, 2001; Buuren & Groothuis-Oudshoorn, 2011). This decision was made to handle the high level of missing data on the dependent variables more effectively since FIML is well-suited for dealing with missing data in longitudinal studies by using all available data points without the need to impute missing values explicitly, thereby reducing potential biases and improving the robustness of the model estimates.

Buuren, S. van, & Groothuis-Oudshoorn, K. (2011). mice : Multivariate Imputation by Chained Equations in R. *Journal of Statistical Software*, *45*, 1‑67. https://doi.org/10.18637/jss.v045.i03

Enders, C. K., & Bandalos, D. L. (2001). The relative performance of full information maximum likelihood estimation for missing data in structural equation models. *Structural Equation Modeling*, *8*(3), 430‑457. https://doi.org/10.1207/S15328007SEM0803_5

**Supp. Table 1.** Bivariate analyses of associations between covariates and the main CLPM outcome scores at baseline (T0).

**Supp. Table 2.** Associations between baseline covariates and outcomes at T1 (linear regression models adjusted for baseline outcome).

**Supp. Table 3.** Comparison of family characteristics at the time of child's autism diagnosis between the mothers’ analysis population (*n* = 315) and those who were excluded (*n* = 561) from the ELENA cohort.

**Supp. Table 4.** Comparison of family characteristics at the time of child's autism diagnosis between the mothers’ complete cases population (*n* = 119) and those who were excluded (*n* = 196) from mothers’ analysis population.

**Supp. Table 5.** Standardized estimates and assessment of Cross-Lagged Panel Models' adequacy for the bidirectional association between mothers' Anxiety/Depression Symptoms and Emotional/Behavioral Difficulties in autistic children following diagnosis.

**Supp. Table 6.** Standardized estimates and assessment of Cross-Lagged Panel Models' adequacy for the bidirectional association between mothers' Anxiety/Depression Symptoms and Internalizing Difficulties in autistic children following diagnosis.

**Supp. Table 7.** Standardized estimates and assessment of Cross-Lagged Panel Models' adequacy for the bidirectional association between mothers' Anxiety/Depression Symptoms and Externalizing Difficulties in autistic children following diagnosis.

**Supp. Table 8.** Standardized estimates and assessment of Cross-Lagged Panel Models' adequacy for the bidirectional association between father's Anxiety/Depression Symptoms (ADS) and Emotional/Behavioral Difficulties (EBD) in autistic children following diagnosis (*n* = 213).

**Supp. Table 9.** Estimations and assessment of Cross-Lagged Panel Models' adequacy for the bidirectional association between mother's Anxiety Symptoms (AS) and Emotional/Behavioral Difficulties (EBD) in autistic children following diagnosis (*n* = 315).

**Supp. Table 10.** Estimations and assessment of Cross-Lagged Panel Models' adequacy for the bidirectional association between mother's Depression Symptoms (DS) and Emotional/Behavioral Difficulties (EBD) in autistic children following diagnosis (*n* = 315).

**Supp. Table 11.** Sensitivity analysis of CLPM: Bidirectional association between mothers' Anxiety/Depression Symptoms and Emotional/Behavioral Difficulties in autistic children following diagnosis adjusted for CBCL version at T0 and T1, and child age at diagnosis (*n* = 315).

**Supp. Table 12.** Sensitivity analysis of CLPM: Bidirectional association between mothers' Anxiety/Depression Symptoms and Internalizing Difficulties in autistic children following diagnosis adjusted for CBCL version at T0 and T1, and child age at diagnosis (*n* = 315).

**Supp. Table 13.** Sensitivity analysis of CLPM: Bidirectional association between mothers' Anxiety/Depression Symptoms and Externalizing Difficulties in autistic children following diagnosis adjusted for CBCL version at T0 and T1, and child age at diagnosis (*n* = 315).

**Supp. Table 14.** Sensitivity analysis of CLPM: Bidirectional association between fathers' Anxiety/Depression Symptoms and Emotional/Behavioral Difficulties in autistic children following diagnosis adjusted for CBCL version at T0 and T1, and child age at diagnosis (*n* = 213).

**Supp. Table 15.** Sensitivity analysis of CLPM: Bidirectional association between mothers' Anxiety Symptoms and Emotional/Behavioral Difficulties in autistic children following diagnosis adjusted for CBCL version at T0 and T1, and child age at diagnosis (*n* = 315).

**Supp. Table 16.** Sensitivity analysis of CLPM: Bidirectional association between mothers' Depression Symptoms and Emotional/Behavioral Difficulties in autistic children following diagnosis adjusted for CBCL version at T0 and T1, and child age at diagnosis (*n* = 315).

**Supp. Table 17.** Clinical comparison of parents’ Anxiety/Depression Symptoms and Emotional/Behavioral Difficulties in autistic children between time of child's autism diagnosis and three years later in matched participants from the mothers’ analysis population.
